# Effect of HIV self-testing on the number of sexual partners among female sex workers in Zambia

**DOI:** 10.1097/QAD.0000000000001740

**Published:** 2018-03-07

**Authors:** Catherine E. Oldenburg, Michael M. Chanda, Katrina F. Ortblad, Magdalene Mwale, Steven Chongo, Nyambe Kamungoma, Catherine Kanchele, Andrew Fullem, Caitlin Moe, Leah G. Barresi, Guy D. Harling, Till Bärnighausen

**Affiliations:** a Francis I. Proctor Foundation; b Department of Ophthalmology; c Department of Epidemiology and Biostatistics, University of California, San Francisco, California, USA; d John Snow, Inc., Lusaka, Zambia; e Department of Global Health and Population, Harvard T.H. Chan School of Public Health; f John Snow, Inc., Boston; g Department of Epidemiology, Harvard T.H. Chan School of Public Health, Boston, Massachusetts, USA; h Research Department of Infection and Population Health, Institute for Global Health, University College London, UK; i Heidelberg Institute of Public Health, University of Heidelberg, Heidelberg, Germany; j Africa Health Research Institute, KwaZulu-Natal, South Africa.

**Keywords:** female sex workers, HIV self-testing, sexual behaviors, sub-Saharan Africa

## Abstract

**Objectives:**

To assess the effect of two health system approaches to distribute HIV self-tests on the number of female sex workers’ client and nonclient sexual partners.

**Design:**

Cluster randomized controlled trial.

**Methods:**

Peer educators recruited 965 participants. Peer educator–participant groups were randomized 1 : 1 : 1 to one of three arms: delivery of HIV self-tests directly from a peer educator, free facility-based delivery of HIV self-tests in exchange for coupons, or referral to standard-of-care HIV testing. Participants in all three arms completed four peer educator intervention sessions, which included counseling and condom distribution. Participants were asked the average number of client partners they had per night at baseline, 1 and 4 months, and the number of nonclient partners they had in the past 12 months (at baseline) and in the past month (at 1 month and 4 months).

**Results:**

At 4 months, participants reported significantly fewer clients per night in the direct delivery arm (mean difference −0.78 clients, 95% CI −1.28 to −0.28, *P* = 0.002) and the coupon arm (−0.71, 95% CI −1.21 to −0.21, *P* = 0.005) compared with standard of care. Similarly, they reported fewer nonclient partners in the direct delivery arm (−3.19, 95% CI −5.18 to −1.21, *P* = 0.002) and in the coupon arm (−1.84, 95% CI −3.81 to 0.14, *P* = 0.07) arm compared with standard of care.

**Conclusion:**

Expansion of HIV self-testing may have positive behavioral effects enhancing other HIV prevention efforts among female sex workers in Zambia.

**Trial Registration:**

ClinicalTrials.gov NCT02827240.

## Introduction

HIV self-testing is a promising strategy to improve HIV testing coverage among diverse populations globally [1,2]. Although HIV self-testing may overcome some traditional barriers to HIV testing, such as stigma or logistical challenges, there may be unintended consequences associated with the use of the test, and these unintended consequences may depend on the strategy for HIV self-testing delivery. Changes in sexual behaviors have been considered following HIV prevention interventions including preexposure prophylaxis [3,4], male circumcision [5], and vaccination [6]. Among MSM, access to HIV self-testing has resulted in increased awareness of HIV risk, which may have led to reduced sexual risk-taking [7,8].

The relationship between HIV self-testing and sexual behaviors is likely complex. We previously demonstrated that HIV self-testing among female sex workers (FSW) in Zambia did not lead to an increase in HIV testing coverage or status knowledge when compared with referral to existing HIV-testing services [9]. Even in the absence of a direct effect of HIV self-testing on status knowledge (which could plausibly lead to sexual behavior change), access to HIV self-testing technology may lead to changes in sexual behaviors. For example, access to HIV self-testing may increase sense of control, as the technology allows freedom of choice of when, where, and with whom to use the test [10]. Furthermore, awareness of and experience with the HIV self-test itself may change perceptions of risk of HIV acquisition, which could lead to changes in sexual behaviors [11]. For example, individuals with access to an HIV self-test may alter their choice of partners if they know they can test themselves or their partner regularly with the self-test [12]. For FSW specifically, access to an HIV self-test may also lead to changes in the price for a sex act. FSW may use the test for themselves or for their clients to generate or demonstrate HIV status information, which could influence the price negotiations. In turn, price differences may lead to changes in income, which could affect the need to sell sex or remain in relationships to sustain a livelihood. Given the potential for these effects, understanding how HIV self-testing influences sexual behaviors is important for the design of HIV self-testing interventions.

Development of effective HIV testing interventions is particularly important among FSW, given their disproportionate burden of HIV [13] and the fact that their engagement in the HIV care cascade remains far below the UNAIDS 90–90–90 targets [14–16]. Here, we report results of a prespecified secondary analysis of the Zambian Peer Educators for HIV Self-Testing (ZEST) study, a three-arm randomized controlled trial of HIV self-testing distribution systems among FSW in Zambia, assessing the overall impact of the HIV self-testing interventions on the number of sex partners.

## Methods

### Participants and procedures

The ZEST study was a three-arm cluster randomized controlled trial comparing two HIV self-test distribution systems to standard-of-care HIV testing for improving HIV testing outcomes among FSW (clinicaltrials.gov NCT02827240). Complete methods for the study have been previously reported [17]. Participants were recruited by trained peer educators in three Zambian transit towns (Livingstone, Chirundu, and Kapiri Mposhi). Participants were eligible if they were at least 18 years of age, reported exchanging sex for money or goods at least once in the previous month, were permanent residents of their town of enrollment, and self-reported that they were not living with HIV, had not recently (<3 months) tested for HIV and did not know their HIV status. Each peer educator recruited an average of six participants.

### Randomization

The randomization unit was the peer educator and the participants she recruited. Peer educator–participant groups were randomized after all participants in the group were enrolled and had completed their baseline assessment. Groups were randomized 1 : 1 : 1 to one of three study arms: distribution of the HIV self-test from the peer educator to her participants (direct delivery), distribution of a coupon from the peer educator to her participants, which could be used to collect an HIV self-test free of charge from one of several distribution points in the town (coupon), or referral to existing HIV-testing options in the town of recruitment by the peer educator. The randomization list was generated in R (Version 3.3.1; The R Foundation for Statistical Computing) in random blocks of 3, 6, and 9 and stratified by town of recruitment. Study assignments for each peer educator were placed in an opaque envelope that was opened by the peer educator and a study staff member, neither of whom knew the assignment beforehand.

### Intervention

In all study arms, peer educators discussed HIV risk reduction strategies, distributed condoms, and referred participants to existing facilities for HIV testing at each peer educator visit. In the direct delivery arm, peer educators distributed two OraQuick ADVANCE Rapid HIV-1/2 Antibody self-tests (OraSure Technologies, Bethlehem, Pennsylvania, USA), one at the first peer educator visit (Week 0) and a second 3 months later at the final peer educator visit (Week 10). In the coupon arm, peer educators distributed two coupons, at weeks 0 and 10, which participants could use to collect an HIV self-test at a participating health clinic or pharmacy. Staff at the health clinics and pharmacies participating in the study received a brief training on the use of the HIV self-test. There were no other changes in the health facilities or pharmacies with regards to staffing, operating hours, or location. All participants had access to a 24-h hotline that they could call if they needed help with HIV testing (including using the test and interpreting the test results), experienced an adverse event such as intimate partner violence, or needed another form of assistance. HIV self-testing was unobserved, as the study was designed to mimic a real-world situation where participants could test at the time and place of their choosing. As such, we did not measure reactions to learning HIV status, although severe adverse psychosocial events were screened for at each study visit.

### Peer educator visits

Participants completed four peer educator intervention visits at weeks 0, 2, 6, and 10. Study assessments occurred at baseline (prior to randomization and the first peer educator visit), at 1 month and at 4 months after the first (Week 0) peer educator visit. Each peer educator visit was conducted following a standardized script. To mimic a real-world peer educator intervention, no study staff were present during peer educator visits. The first peer educator visit in all arms was a group visit; subsequent visits were one-on-one. All peer educator visits in all arms consisted of HIV risk reduction counseling, information on where to go for HIV testing, and provision of condoms. In the interventions, peer educators additionally provided a brief training on the use of HIV self-tests and distributed either the HIV self-test kits or the coupons.

### Measures

All assessments were completed via computer-assisted personal interviewing at three study assessments at the time of enrollment, and at months 1 and 3 following the first peer educator visit. Prior to randomization, participants completed a baseline questionnaire, which covered several domains, including sociodemographic characteristics (age, literacy, educational attainment, mobile phone ownership, monthly income) and sex work history (including the age at which they first started working in sex work, how often condoms were available to them while working and how much they cost, and how much they normally charged for vaginal sex). Having a primary partner was defined as a stable, noncommercial partner such as a husband or boyfriend.

Sexual behaviors with clients and nonclient partners were measured at baseline, 1, and 4 months. To measure sexual behaviors with clients, participants were asked on average how many client sexual partners they had per night. At baseline, participants were asked how many nonclient partners they had in the previous 12 months. At 1 and 4 months, participants were asked how many nonclient partners they had had in the previous month. At 1 and 4 months, participants were asked when their last HIV test was, and the result of this last test.

### Statistical methods

All analyses were intention-to-treat. Prespecified outcomes included the average number of client and nonclient partners at 1 and 4 months. We used a mixed-effects generalized linear model with a Gaussian distribution to estimate the mean difference in number of partners (client and nonclient) at each time point by study arm. Each model included a fixed effect for randomization arm, study site, and baseline (e.g. baseline average number of partners) and a random effect for peer educator group. As per our prespecified analysis plan, all analyses were carried out as complete case analyses. All tests were two-sided with no adjustments for multiple comparisons. All analyses were conducted in Stata 14.1 (StataCorp, College Station, Texas, USA).

## Results

**Fig. 1:**
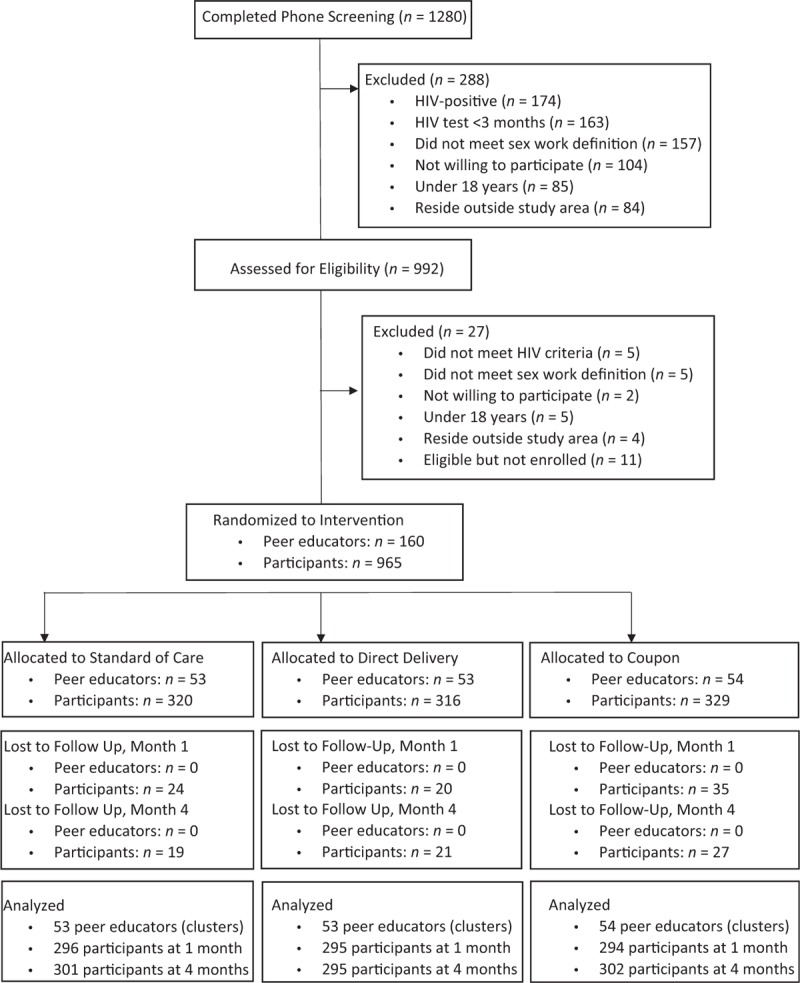
CONSORT flow diagram of screened, randomized, and analyzed participants.

From September to October 2016, 1280 potential participants were screened and 965 were found eligible and subsequently enrolled in the study (Fig. 1). A mean of six participants in 160 peer educator groups were randomized to one of the three study arms. Baseline characteristics were similar between the three groups (Table 1). Participants were a median of 25 years of age [interquartile range (IQR) 21–30] and participants had been engaging in sex work for a median of 5 years (IQR 3–10 years). Few participants had regular access to free condoms while working. Missing sexual behaviors data were uncommon. At 1 month, 877 of 885 (99.1%) of participants who were retained in the study had complete data related to sexual partners. At 4 months, 891 of 898 (99.2%) had complete sexual partners’ data. At 1 month, 89.3% of participants reported testing for HIV in the previous month, and 79.6% of participants at 4 months reported testing in the previous month [9]. By 4 months, only a single participant reported that they had never tested for HIV. At 1 month, 144 (16.5%) of participants reported that their most recent HIV test was positive, which increased to 235 (26.4%) at 4 months.

**Fig. 2:**
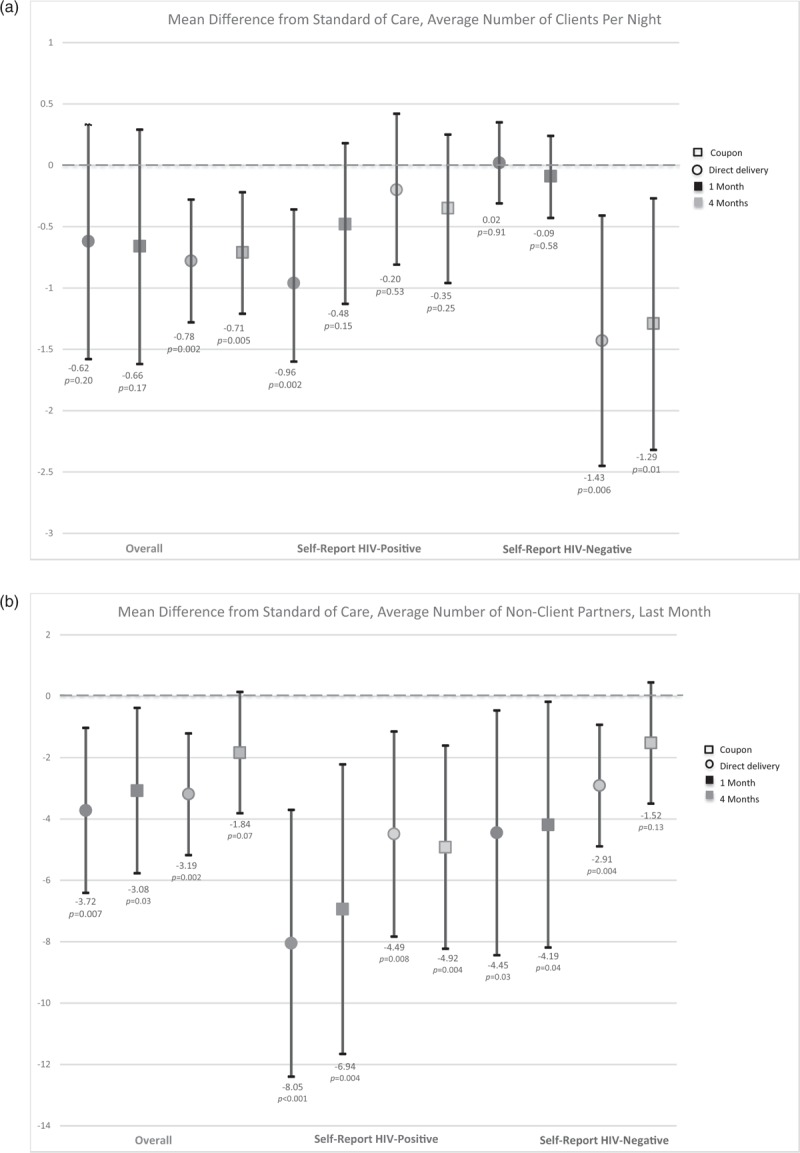
Mean difference in number of client (a) and nonclient (b) partners at each time point in the direct delivery (circle) and coupon (square) arms, overall (red) and by self-reported HIV status (negative = green; positive = blue).

At baseline, the mean number of clients on an average night were reported to be 5.3 (SD 7.5) in the standard-of-care HIV testing arm, 4.6 (SD 8.8) in the direct delivery arm, and 4.2 (SD 5.7) in the coupon arm. The number of clients decreased over time in all arms from baseline to 1 and to 4 months (Table 2; Fig. 2a). Although there was no difference across arms in mean number of clients at 1 month compared with the standard-of-care arm, at 4 months participants reported significantly fewer clients per night in the direct delivery arm (mean difference −0.78 compared with the standard-of-care arm, 95% CI −1.28 to −0.28, *P* = 0.002) and the coupon arm (mean difference −0.71 compared with the standard-of-care arm, 95% CI −1.21 to −0.21, *P* = 0.005). This difference was primarily driven by individuals who reported a negative HIV test at 4 months. There was no difference in the number of clients among participants who reported testing positive for HIV at 4 months, but participants in the direct delivery arm who reported a positive HIV test at 1 month reported significantly fewer clients per night at 1 month compared with the standard-of-care arm (mean difference −0.96, 95% CI −1.6 to −0.36, *P* = 0.002).

Darker shading indicates 1-month time point and lighter shading indicates the 4-month time point. Red indicates the overall effect, green among individuals self-reporting an HIV-positive status at their last HIV test, and blue an HIV-negative status at their last HIV test. Black bars indicate 95% confidence intervals for the difference from the standard-of-care arm. For each category, the first two point estimates indicate the 1-month effects (darker shading) and the second two indicate the four-month effects (lighter shading).

At baseline, participants reported a mean of 17.2 (SD 34.7) nonclient sexual partners over the previous year in the standard-of-care arm, 18.8 (SD 54.3) in the direct delivery arm, and 19.5 (SD 64.4) in the coupon arm. In the direct delivery arm, participants reported significantly fewer nonclient partners over the previous 30 days at 1 month (mean difference −3.72, 95% CI −6.41 to −1.03, *P* = 0.007) and at 4 months (mean difference −3.19, 95% CI −5.18 to −1.21, *P* = 0.002; Fig. 2b) compared with the standard-of-care arm. In the coupon arm, the number of nonclient partners decreased at 1 month compared with the standard-of-care arm (mean difference −3.08, 95% CI −5.77 to −0.38, *P* = 0.03) but not at 4 months (mean difference −1.84, 95% CI −3.81 to 0.14, *P* = 0.07; Fig. 2b). Both participants who reported testing negative and positive for HIV at each time point reported fewer nonclient partners in the past month in the direct delivery and coupon arms compared with the standard-of-care arm. Participants reporting that they were HIV-positive at their most recent test reported a larger decrease in the number of partners in the direct delivery and coupon arms relative to the standard-of-care arm than those who tested HIV-negative (Fig. 2b).

## Discussion

In this study of HIV self-testing among FSW in Zambia, access to an HIV self-test led to a significant decrease in the overall number of client and nonclient sexual partners. This may have occurred via several mechanisms, independent of acquisition of knowledge of one's own HIV status. As HIV self-testing is a user-controlled intervention, and individuals can use it to test in the time and place of their choice, it may increase perception of control over one's situation. Increasing a sense of agency or empowerment through HIV self-testing may result in participants changing the number of sexual partners [18].

Women frequently enter sex work because of financial pressures [19], and there are financial incentives for having multiple client partners. Women may have gained demonstrable proof of negative HIV status by self-testing and as a result were able to charge higher prices per sex act than without such proof. In turn, the higher prices per sex act could reduce the number of clients, if FSW have a target income [20]. Given that effects were stronger and more consistent among nonclient partners, the observed change in sexual partners may not be exclusively driven by economic concerns, although nonclient partners often offer housing or other monetary assistance. Women may have reduced their number of partners because they were afraid of partners seeing the test and they had no place to store it. Although no serious adverse events related to learning HIV status were reported, we cannot rule out that some women have had strong emotional reactions to learning their status, which may have influenced behavior or psychosocial well-being leading to fewer sex partners.

Most participants tested for HIV in the previous month, and there was no difference in past-month HIV testing at either time point by arm [9]. Although the reduction in the number of client sexual partners at 4 months was primarily driven by participants who reported that their most recent HIV test was negative, the number of nonclient sexual partners for participants reporting both negative and positive test results decreased. For individuals testing HIV-negative, HIV self-testing may enhance HIV prevention efforts both by being another option for frequent re-testing and also by effecting positive change on sexual behaviors. The reduction in client sexual partners may reflect a desire for FSWs to maintain HIV-negative status. For those testing HIV-positive, HIV self-testing may enhance positive prevention leading to a reduction in the number of partners. The reduction in the number of nonclient sexual partners among those testing HIV-positive might reflect a desire to avoid transmitting HIV to primary or other nonclient partners. However, our results are unlikely to be primarily driven by learning HIV status.

A concern with HIV self-testing, which has been discussed in the literature, is that sexual risk-taking behavior could increase following a negative test [1,21,22]. In the present study, we found no evidence that a negative test would lead to increases in sexual risk behavior. On the contrary, participants reporting a negative test reported fewer clients and nonclient partners overall. Previous studies of sexual behavior changes following HIV self-testing have overwhelmingly been in MSM in high-income countries [1,7,8,23,24]. These studies generally demonstrate that HIV self-testing increases awareness of risk and thus, leads to reduced sexual risk. However, behavioral patterns following HIV self-testing may differ dramatically for FSW in settings such as Zambia. A qualitative study among FSW in Kenya who distributed HIV self-tests to partners demonstrated that HIV self-testing affected informed sexual decision-making with both client and nonclient partners [12]. HIV self-testing allowed women to know that both they and their primary partner were HIV-negative and that they would thus, not need to use condoms. Knowledge of a client's positive HIV status led participants to discontinue relationships with that client [12]. In the present study, participants did not distribute HIV self-tests to their partners: thus, the intervention only led to awareness of the participant's own HIV status. In combination with evidence from previous studies, the results of this study do not provide evidence that HIV self-testing increases sexual risk behavior. Rather our findings indicate that HIV self-testing may substantially reduce sexual risk-taking and HIV acquisition.

The results of this study must be considered in the context of some limitations. Participants in all arms received a peer educator intervention, which may have masked some effects on sexual behaviors. However, the intervention was randomly assigned and all participants, including those in the standard-of-care arm, received the identical peer educator intervention. We did not collect data on potential mechanisms driving the reduction in partners seen in this study. The precise mechanisms for the effects are unknown. Qualitative and quantitative research is needed to elucidate which of the several plausible pathways from HIV self-testing to the number of sex partners transmit the effects shown in this study. Such mechanistic insights will be useful in designing future HIV self-testing initiatives and accompanying interventions. Finally, all outcome measures relied on self-report, including sexual behaviors and results of the most recent HIV test. Social desirability bias may have led participants to underreport risky sexual behaviors or positive HIV tests results. However, such bias is unlikely to occur differentially across arms of this randomized trial. As such, we anticipate that any effect of social desirability would bias results towards the null, so that our findings are conservative in estimating sex partner reductions because of the HIV self-testing interventions.

We found no evidence of increases in sexual risk-taking behavior following HIV self-testing in this randomized controlled trial among FSW in Zambia. On the contrary, FSW in HIV self-testing arms of the study reported significantly fewer client and nonclient partners. In this setting in Zambia, sexual behavior changes among FSW following HIV self-testing does not appear to be a concern, and thus, should not limit expansion of HIV self-testing programs. There may be positive behavioral benefits of HIV self-testing related to reduced HIV risk-taking among FSW.

## Acknowledgements

We gratefully acknowledge the research assistants who collected data for this study, the members of the Scientific Oversight Committee, the peer educators, and the participants who dedicated their time to this study.

Funding: The Zambian Peer Educators for HIV Self-Testing (ZEST) study was funded by the International Initiative for Impact Evaluation (3ie). C.E.O. was supported in part by the National Institute on Drug Abuse T32-DA013911 and the National Institute of Mental Health R25-MH083620. K.F.O. was supported in part by the National Institute of Allergy and Infectious Disease T32-AI007535. T.B. was funded by the Alexander von Humboldt Foundation through the Alexander von Humboldt Professorship endowed by the German Federal Ministry of Education and Research. He was also supported by the Wellcome Trust, the European Commission, the Clinton Health Access Initiative and NICHD of NIH (R01-HD084233), NIAID of NIH (R01-AI124389 and R01-AI112339) and FIC of NIH (D43-TW009775). OraQuick HIV self-tests were obtained from the manufacturer at cost. The study sponsor was not involved in study design, collection, management, analysis or interpretation of the data, of writing reports, or in the decision to submit the report for publication.

### Conflicts of Interest

There are no conflicts of interest.

## Figures and Tables

**Table 1:** Baseline descriptive characteristics by randomization arm.

	Standard of Care (*N* = 320)	Direct HIVST delivery (*N* = 316)	HIVST coupon (*N* = 329)
Age (median, IQR)	25 (22–31)	25 (21–30)	25 (21–30)
Site			
Livingstone	156 (48.8%)	162 (51.3%)	162 (49.2%)
Kapiri	87 (27.2%)	76 (24.1%)	82 (24.9%)
Chirundu	77 (24.1%)	78 (24.7%)	85 (25.8%)
Have a primary partner	203 (63.6%)	171 (54.1%)	202 (61.0%)
Can read and write	226 (70.9%)	243 (77.1%)	253 (77.9%)
Education			
No formal education	53 (16.6%)	30 (9.5%)	25 (7.5%)
Primary/junior	129 (40.3%)	152 (48.1%)	169 (51.5%)
Secondary	131 (40.9%)	128 (40.5%)	130 (39.6%)
Vocational	6 (1.9%)	6 (1.9%)	1 (0.3%)
Tertiary	1 (0.3%)	0	3 (0.9%)
Mobile phone ownership	271 (84.7%)	265 (83.9%)	284 (86.3%)
Monthly income			
No income	81 (25.8%)	58 (18.7%)	63 (19.4%)
<250 kwacha^a^	40 (12.7%)	32 (10.3%)	51 (15.7%)
251–500 kwacha^a^	75 (23.9%)	86 (27.7%)	74 (22.8%)
501–1000 kwacha^a^	74 (23.6%)	82 (26.4%)	90 (27.8%)
1001–1500 kwacha^a^	17 (5.4%)	30 (9.7%)	26 (8.0%)
>1500 kwacha^a^	27 (8.6%)	23 (7.4%)	20 (6.2%)
Years in sex work (median, IQR)	5 (3 to 10)	5 (3 to 10)	5 (3 to 8)
Condom availability while working			
Never	4 (1.3%)	5 (1.6%)	8 (2.4%)
Seldom/sometimes	225 (70.5%)	217 (68.9%)	206 (62.6%)
Often/always	90 (28.2%)	93 (29.5%)	115 (35.0%)
Condoms available for free while working, often/always	45 (14.4%)	47 (15.0%)	53 (16.4%)
Number of clients on an average night (mean, SD)	5.3 (7.5)	4.6 (8.8)	4.2 (5.7)
Inconsistent condom use with clients	231 (75.2%)	236 (78.7%)	228 (71.0%)
Number of nonclient partners in the past 12 months (mean, SD)	17.2 (34.7)	18.8 (54.3)	19.5 (64.4)
Inconsistent condom use with nonclients	229 (74.8%)	209 (69.4%)	216 (69.5%)

HIVST, HIV self-testing; IQR, inter-quartile range.

^a^50 Zambian kwacha = approximately US$1.

**Table 2:** Average number of clients at each time point by randomization arm.

	Average number of clients per night (SD)	Average number of nonclient partners, last month (SD)
One month		
Standard of care	4.6 (8.9)	7.8 (21.3)
Direct delivery	3.9 (2.2)	3.8 (9.7)
Coupon	3.8 (2.7)	4.6 (9.1)
Four months		
Standard of care	4.3 (3.9)	6.5 (13.6)
Direct delivery	3.5 (1.7)	2.8 (5.0)
Coupon	3.6 (1.6)	4.2 (7.2)
Self-reported HIV-positive		
One month		
Standard of care	4.6 (2.5)	9.9 (16.4)
Direct delivery	3.5 (1.3)	2.2 (4.0)
Coupon	3.9 (1.9)	2.8 (3.0)
Four months		
Standard of care	4.1 (1.8)	8.6 (17.4)
Direct delivery	3.7 (2.1)	2.6 (4.2)
Coupon	3.7 (1.6)	4.1 (6.8)
Self-reported HIV-Negative		
One month		
Standard of care	4.0 (2.7)	6.3 (22.5)
Direct delivery	3.9 (1.6)	3.3 (4.6)
Coupon	3.7 (1.5)	3.6 (5.0)
Four months		
Standard of care	4.4 (4.6)	5.8 (12.0)
Direct delivery	3.4 (1.5)	2.8 (5.2)
Coupon	3.5 (1.4)	4.0 (7.1)
